# Ischial tuberosity avulsion fractures: limited evidence supporting displacement cut-offs for surgery

**DOI:** 10.1308/rcsann.2025.0098

**Published:** 2025-10-07

**Authors:** HD Veldman, RM Jeuken, TAG van Vugt, L Verlaan

**Affiliations:** ^1^ Zuyderland Medical Centre, Sittard-Geleen, The Netherlands; ^2^ Maastricht University Medical Centre+, The Netherlands

We read with great interest the article by Shaharudin and colleagues on the management of ischial tuberosity avulsion fractures (ITAF) among hip surgeons in the UK.^1^ Their survey underscores the lack of consensus, and we agree that the current evidence is too limited and heterogeneous to underpin definitive treatment guidelines.

The study highlights the central role of displacement thresholds: among those considering surgery, 61.5% regarded ≥20mm, 19.2% regarded ≥15mm and 19.2% regarded any displacement as significant.^1^ In previous literature, the 15 and 20mm thresholds are frequently cited as cut-offs to justify operative intervention. Shaharudin *et al* pointed out that the so-called ‘grey area’ lies between 15 and 20mm, where both surgical and conservative approaches may yield favourable outcomes.^1^

In our recent publication, we extensively reviewed the origins and epidemiological basis of these thresholds and found the data too limited to justify a definitive surgical cut-off.^2^ In brief, the key findings from our review can be summarised as follows:

• The commonly cited 20mm threshold can mainly be traced back to Schuett *et al*, who reviewed 228 pelvic apophyseal avulsion fractures.^3^ They reported a higher risk of non-union when displacement exceeded 20mm, with four non-unions being ITAF cases, and just one of these patients developed symptoms.^3^ Notably, this symptomatic non-union had an initial displacement of only 6mm, which challenges the validity of using 20mm as a rigid operative indication in ITAF.^3^

• The 15mm threshold is mainly linked to the small cohort reported by Ferlic *et al.* Of 13 patients, 9 had displacements >15mm.^4^ Five patients underwent surgical fixation with excellent results, and four were managed conservatively; two of these four (50%) developed pseudoarthrosis and reported pain when sitting and running for a longer period.^4^ All four patients with <15mm displacement did excellently with conservative management. On this limited basis, the authors recommended conservative treatment below 15mm and early surgery in active patients with greater displacement.^4^

• These cases reported by Ferlic *et al* have subsequently carried disproportionate weight in the literature, as in the widely cited meta-analysis by Eberbach *et al* they were the only conservatively treated patients with >15mm displacement.^4,5^ In that subset, outcomes appeared less favourable than in surgically treated cases (50% vs 84% excellent results), which the authors interpreted as support for recommending surgery in patients with >15mm displacement and high functional demands.^5^

Therefore, we consider these findings to be not epidemiologically robust enough to serve as definitive guidelines and should therefore be applied with caution. It is also important to note that fractures with minimal initial displacement may develop persistent symptoms, and secondary displacement and non-union of an initially minimally displaced fragment can occur.^3,6,7^ Conservative treatment with close follow-up may be reasonable in minimally displaced cases, but delayed surgery is technically more demanding, often requiring extensive sciatic nerve dissection and more complex fixation. For young active patients, prolonged symptoms before eventual surgery may also be undesirable. Moreover, although limited data availability and selection bias must be considered, current evidence indicates that operatively treated ITAF patients generally achieve higher success rates, better return-to-sport rates, and fewer complications than those managed non-operatively.^5,8^

In conclusion, although displacement remains an important factor in decision making, the evidence supporting commonly cited thresholds remains weak. We advocate for an individualised, patient-centred approach in which functional goals, risks, and expectations are carefully weighed to guide decision making (see figure 6 in our article for primary considerations).^2^ At the same time, there is a need for further research, with structured reporting of treatment strategies and outcomes to help shape more reliable future treatment protocols.

## Competing interests

The authors declare no conflict of interest.

## Funding

The authors received no financial support for the research, authorship, and/or publication of this article.

## Ethics statement

Not applicable.

## Author contributions

**HD Veldman:** Conceptualisation, Writing – original draft. **RM Jeuken:** Conceptualisation, Writing – review & editing. **TAG van Vugt:** Conceptualisation, Writing – review & editing. **L Verlaan:** Conceptualisation, Writing – review & editing.

## Artificial Intelligence

The author/s declare that no AI was used to conduct the study or prepare the manuscript.
